# Crystal Growth and Spectroscopic Investigations of Tm^3+^:Li_3_Ba_2_Gd_3_(MoO_4_)_8_ Crystal

**DOI:** 10.3390/ma7010496

**Published:** 2014-01-17

**Authors:** Mingjun Song, Lintong Wang, Nana Zhang, Xishi Tai, Guofu Wang

**Affiliations:** 1 School of Chemistry and Chemical Engineering, Weifang University, Weifang 261061, Shandong, China; E-Mails: wanglt6408@sina.com (L.W.); zhangnana0801@126.com (N.Z.); taixs@wfu.edu.cn (X.T.); 2 Fujian Institute of Research on the Structure of Matter, The Chinese Academy of Sciences, Fuzhou 350002, Fujian, China; E-Mail: wgf@fjirsm.ac.cn

**Keywords:** crystal growth, optical materials, spectral properties, cross section

## Abstract

Tm^3+^:Li_3_Ba_2_Gd_3_(MoO_4_)_8_ crystal has been grown by the top seeded solution growth (TSSG) method from a Li_2_MoO_4_ flux. The room temperature polarized absorption spectra, fluorescence spectra, and fluorescence decay curves of the crystal were measured. Based on the Judd-Ofelt (J-O) theory, the main spectroscopic parameters of the crystal, including the spontaneous emission probabilities, fluorescence branching ratios, and radiative lifetimes were calculated and analyzed. The broad and strong absorption bands of the crystal show that it can be efficiently pumped by the diode laser, while the large emission cross-sections of the ^3^F_4_ → ^3^H_6_ transition indicate that the crystal is a promising candidate for tunable and short pulse lasers.

## 1. Introduction

With the rapid progress of high power diode lasers, Tm^3+^-doped laser media has been intensively investigated because of their potential applications associated with emissions in the visible and infrared spectral regions, particularly at ~1.50 and ~2.0 μm wavelength. As we know, the ^3^F_4_ → ^3^H_6_ transition of Tm^3+^ ions is one of the most effective channels for the development of 2.0 μm lasers, which have many important applications in fields of medicine, gas detection, remote sensing, *etc*. On the other hand, the ^3^H_4_ → ^3^F_4_ transition of Tm^3+^ ions gives rise to an additional infrared emission band around 1.5 μm, where applications such as detection, ranging and optical communication have been found. Other important advantages of Tm^3+^ ions include strong absorption of AlGaAs diode laser radiation at ~800 nm, a long lifetime of the ^3^F_4_ state, a high quantum efficiency due to the cross-relaxation between ^3^H_4_ and ^3^F_4_ multiplets, as well as a wide emission band around in the range of 1800–2000 nm, which is definitely promising for the generation of tunable and ultrafast solid state lasers. To date, efficient laser operations around 2.0 μm have been realized in a number of Tm^3+^-doped crystals [1–10].

Among the reported crystals, the double tungstate and molybdate crystals with scheelite structure are characterized by their local disordered crystal structure [4–8]. The advantages of these crystals are high quantum efficiency, broad absorption and emission brands, as well as relatively low upper-level lifetime. Furthermore, such a combination of these merits is very promising for generations of tunable and ultra fast lasers. More recently, a new series of disordered molybdate compounds Li_3_Ba_2_Re_3_(MoO_4_)_8_ (Re = La-Lu, Y), which belong to the monoclinic system, with the space group C2/c, have emerged as new kinds of laser materials, especially in tunable and ultrafast laser domains [11–17]. The structure of Li_3_Ba_2_Re_3_(MoO_4_)_8_ can also be considered to be derived from the scheelite structure, in which the Ca^2+^ sites are occupied by a statistical mixture of 25% Ba^2+^, 37.5% Li^+^, and 37.5% Re^3+^ [13]. Consequently, rare earth doped Li_3_Ba_2_Re_3_(MoO_4_)_8_ crystals usually exhibit very similar spectral properties to the typical disordered double molybdate crystals. Up to now, efficient laser operation has already been realized in Nd^3+^ and Yb^3+^-doped Li_3_Ba_2_Gd_3_(MoO_4_)_8_ crystals [11,13]. Not long ago, Zaldo *et al*. [16] have demonstrated the great potential of Tm^3+^:Li_3_Ba_2_Lu_3_(MoO_4_)_8_ crystal for tunable and ultrashort pulse lasers around 2 μm, *i.e.*, a tunable laser in the range of 1853–2009 nm, with a slope efficiency up to 71%, higher than those obtained in disordered crystals so far, has been obtained with a Ti:laser as pump source. Furthermore, the large free running laser bandwidth indicated that the Tm^3+^:Li_3_Ba_2_Lu_3_(MoO_4_)_8_ crystal is definitely promising for generations of ultrafast laser pulses. In the present work, the studies are extended to Tm^3+^-doped Li_3_Ba_2_Gd_3_(MoO_4_)_8_ crystal with the objective of exploring new Tm^3+^-doped crystal for efficient laser operations near 2 μm, and the growth and spectral properties of Tm^3+^:Li_3_Ba_2_Gd_3_(MoO_4_)_8_ crystal are reported.

## 2. Experimental Section

### 2.1. Crystal Growth and Orientation

**Figure 1.**
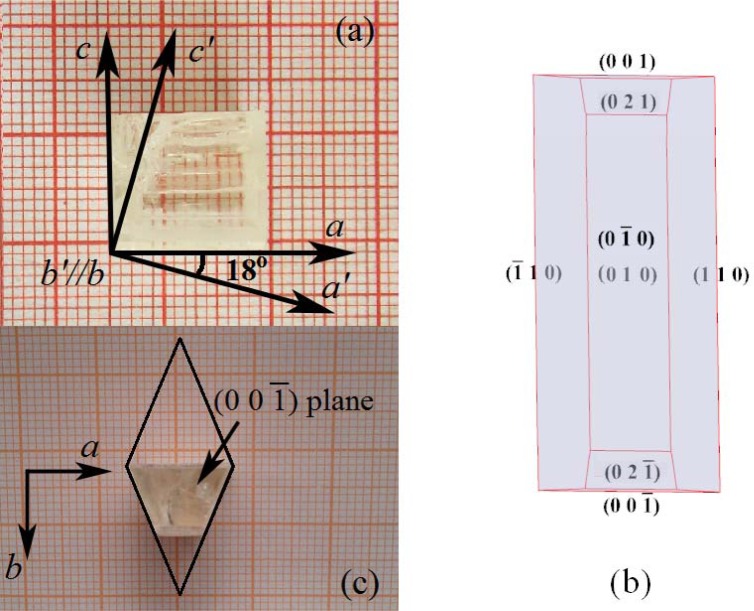
(**a**) Grown crystal of Tm^3+^:Li_3_Ba_2_Gd_3_(MoO_4_)_8_; (**b**) The morphological scheme of the crystal; (**c**) Cross-section of the as grown crystal.

As Li_3_Ba_2_Gd_3_(MoO_4_)_8_ crystal melts incongruently [12], the crystal was grown by the top seeded solution growth (TSSG) method from a flux of Li_2_MoO_4_. The solubility curve of Li_3_Ba_2_Gd_3_(MoO_4_)_8_ crystal in the Li_3_Ba_2_Gd_3_(MoO_4_)_8_-Li_2_MoO_4_ solution can be found in Reference [14]. The crystal growth was carried out in a vertical tubular muffle furnace with a nickel–chrome wire as the heating element. An AL-708 apparatus controlled the furnace temperature and the rate of cooling. The starting materials, 3 at% Tm^3+^-doped Li_3_Ba_2_Gd_3_(MoO_4_)_8_ and Li_2_MoO_4_ were weighed, with a molar ratio of TmLi_3_Ba_2_Gd_3_(MoO_4_)_8_:Li_2_MoO_4_ = 1:4. A single-crystalline bar cut along *b*-direction was used to introduce crystal nucleation. The growth temperature interval was 900–870 °C, with a cooling rate of 1 °C/d and a rotating rate of 12 rpm. Additional details on crystal growth can be found in Reference [14,15]. The as-grown Tm^3+^:Li_3_Ba_2_Gd_3_(MoO_4_)_8_ crystal with a prism shape is shown in Figure 1a. The initial dimension of the grown crystal is about 15 × 10 × 40 mm^3^ along the *a*, *b*, and *c*-directions, respectively. However, due to the cleavable nature of the crystal, the grown crystal has split along the middle along the cleavage plane (001) during the annealing process. As a result, only a small part of the obtained crystal is shown in Figure 1a. Figure 1b shows the morphological scheme of the grown crystal. The rectangular facets on the top and bottom of the crystal were established to be (010) and (0−10) faces, respectively, which are consistent with those of the Li_3_Ba_2_Lu_3_(MoO_4_)_8_ crystal [17]. However, different from the Li_3_Ba_2_Lu_3_(MoO_4_)_8_ crystal, the facets along sides of the crystal were identified to be (110) and (−110) faces, respectively, rather than (100) and (−100) faces. As a consequence, the grown crystal possesses a trapezoid cross-section, as shown in Figure 1c. It can be found that the profile of the (00−1) face of the Tm^3+^:Li_3_Ba_2_Gd_3_(MoO_4_)_8_ crystal is in good accordance with that of the Yb^3+^:Li_3_Ba_2_Gd_3_(MoO_4_)_8_ and Er^3+^:Li_3_Ba_2_Y_3_(MoO_4_)_8_ crystal grown along the *a*-direction [12,15].

As the Li_3_Ba_2_Gd_3_(MoO_4_)_8_ crystal is biaxial, the three principal axes of the optical indicatrix should be determined before the polarized spectral measurement. In the monoclinic system, one of the principal axes is collinear with the crystallographic *b*-axis and the other two principal axes are perpendicular and positioned at certain angles with respect to crystallographic *a* and *c*-axes. Then, the other two principal axes can be easily determined when the sample is viewed along the *b*-axis direction under a polarized microscope. However, due to the restriction of the test conditions, the precise values of the refractive index for the three principal axes were not measured. As the cell parameters and the positions of the three principal axes relative to the crystallographic axes are very close to the Li_3_Ba_2_Lu_3_(MoO_4_)_8_ crystal, the three principal axes are temporarily named in the same way as the Li_3_Ba_2_Lu_3_(MoO_4_)_8_ crystal [16,17]. As shown in Figure 1a, the principal axis collinear with the crystallographic *b* axis is named as *b*′; *a*′ is rotated at 18° with respect to *a* axis in the clockwise direction as the crystals are viewed from the negative *b* axis, finally, *c*′ is perpendicular to *a*′ and *b*′.

### 2.2. Characterizations

The polarized absorption spectra were measured using a Perkin–Elmer UV-VIS-NIR spectrometer (Lambda-900) in a wavelength range of 300–2000 nm, the wavelength resolution of which is 0.08 nm. The polarized fluorescence spectra and the fluorescence decay curves were measured using an Edinburgh Instruments FLS920 spectrophotometer, the wavelength resolution of which is 0.2 nm. The concentration of Tm^3+^ ions in the Tm^3+^:Li_3_Ba_2_Gd_3_(MoO_4_)_8_ was measured to be 1.26 at% by the ICP atomic emission spectroscopy analysis method. Thus, the corresponding concentration and the segregation coefficient of Tm^3+^ ions in the grown crystal were 6.3 × 10^−19^ cm^2^ and 0.42, respectively. All the above measurements were carried out at room temperature.

## 3. Results and Discussion

### 3.1. Absorption Spectra and Judd-Ofelt Analysis

**Figure 2.**
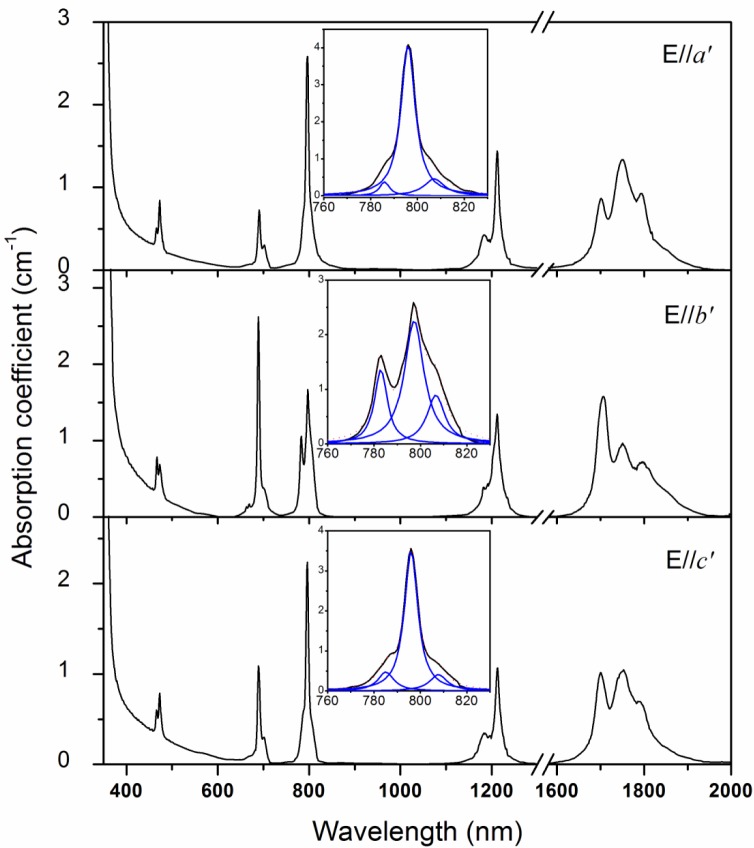
Polarized absorption spectra of Tm^3+^:Li_3_Ba_2_Gd_3_(MoO_4_)_8_ crystal.

Figure 2 shows the polarized absorption spectra of Tm^3+^:Li_3_Ba_2_Gd_3_(MoO_4_)_8_ crystal measured at room temperature. For all polarizations, strong absorption bands occur around 475, 690, 800, 1200, and 1750 nm, corresponding to the transitions of Tm^3+^ ions from the ground state ^3^H_6_ to the excited states ^1^G_4_, ^3^F_2,3_^3^H_4_, ^3^H_5_, and ^3^F_4_, respectively. The most attractive band of the absorption spectra is the one around 800 nm, which belongs to the ^3^H_6_ → ^3^H_4_ transition and is the main pumping channel for Tm^3+^ ions. The inset of Figure 2 shows the absorption cross-sections σ_abs_ of this band for clarity. As this band is composed of several overlapped peaks, a Lorentz fit was applied to it and three peaks, around 785, 796, and 805 nm, were found. For all polarizations, the peak absorption cross-sections are located at 796 nm and the values are 4.08, 2.59, and 3.55 × 10^−20^ cm^2^ for E//*a*′, E//*b*′, and E//*c*′, respectively. The full width at half the maximum (FWHM) of the 796 nm absorption peaks are 9, 11, and 8 nm for E//*a*′, E//*b*′, and E//*c*′, respectively, which are close to those of other disordered molybdate crystals, such as Tm^3+^:LiLa(MoO_4_)_2_ (8 nm for σ polarization) [6], Tm^3+^:LiGd(MoO_4_)_2_ (8 nm for σ polarization) [8], and Tm^3+^:Ba_2_Gd_4_(MoO_4_)_4_ (7 nm for E//X and 8 nm for E//Y) [7], but much larger than those of ordered Tm^3+^:YAG (4 nm) [1], Tm^3+^:YVO_4_ (5 nm for π polarization) [2], and Tm^3+^:KY(WO_4_)_2_ (5.4 nm for E//N_m_) crystals [3]. Such a broad bandwidth indicates an inhomogeneous broadening behavior of Tm^3+^:Li_3_Ba_2_Gd_3_(MoO_4_)_8_ crystal and make the crystal very suitable for diode pumping, as the thermal stabilization of pumping source is not so critical in this case. Furthermore, it can be found that both the profile and the intensity of the absorption bands of Tm^3+^:Li_3_Ba_2_Gd_3_(MoO_4_)_8_ are similar to those of the isostructural Tm^3+^:Li_3_Ba_2_Lu_3_(MoO_4_)_8_ crystal [16].

The Judd-Ofelt (J-O) theory has been widely used in the analysis of the spectroscopic properties of rare-earth ions in crystals and glasses [18,19] According the J-O theory, the Judd-Ofelt parameters Ω_t_ (*t* = 2, 4, 6) for each polarization can be calculated by a least-square fitting between the theoretical and the experimental line strengths for the electric-dipole transitions. Then, the spontaneous emission probabilities of the electric-dipole *A*^ed^ and magnetic-dipole *A*^md^ transitions, the fluorescence branching ratios β and the radiative lifetime τ_r_ can be estimated. In the present work, only the calculated results are presented, and the detailed calculation procedures are similar to those reported in Reference [4]. The values of the refractive index, n, at different wavelengths were calculated using the Sellmeier Equations:

$${\text{n}}^{2}=\text{A}+\frac{\text{B}\lambda^{2}}{\lambda^{2}-{\text{C}}^{2}}-\text{D}\lambda^{2}$$ (1)

where the constants *A*, *B*, *C*, and *D* for the three polarizations were derived from Reference [17].

The values of *A*^ed^, *A*^md^, β, and τ_r_ of some typical transitions are listed in Table 1.

### 3.2. Fluorescence Spectra and Emission Cross-Sections

**Figure 3.**
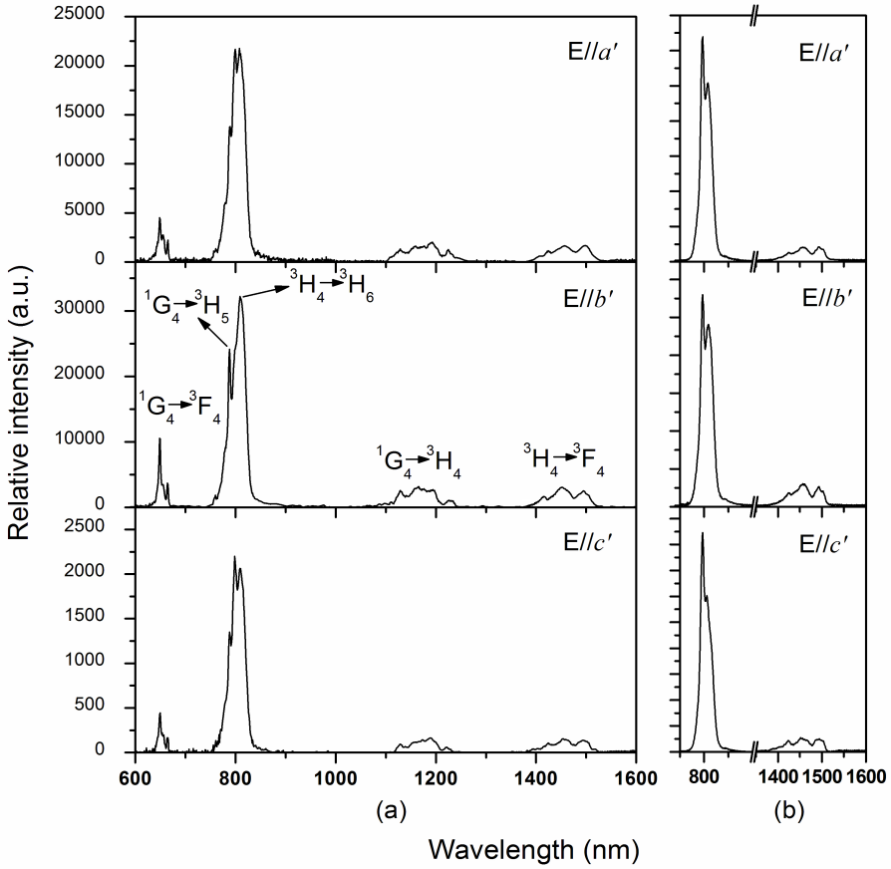
Polarized fluorescence spectra of Tm^3+^:Li_3_Ba_2_Gd_3_(MoO_4_)_8_ crystal: (**a**) Excited with 473 radiation; (**b**) Excited with 688 nm radiation.

Figure 3 shows the polarized emission spectra of Tm^3+^:Li_3_Ba_2_Gd_3_(MoO_4_)_8_ crystal in a range of 600–1600 nm, when the samples were excited into the ^1^G_4_ state with 475 nm radiation. There are four main emission bands around 650, 800, 1175, and 1450 nm for each polarization. The emission bands around 650, 1175, and 1450 nm can be assigned to the ^1^G_4_ → ^3^F_4_, ^1^G_4_ → ^3^H_4_, and ^3^H_4_ → ^3^F_4_ transitions, respectively. The emission band around 800 nm should be the superposition of two resonant transitions, namely ^1^G_4_ → ^3^H_5_ and ^3^H_4_ → ^3^H_6_, which were severely overlapped due to the very close barycentric wavelengths. To confirm the conclusion, the polarized emission spectra were also measured under 688 nm excitation, *i.e.*, the Tm^3+^ ions were excited to the ^3^F_2,3_ states. In this case, the ^3^H_4_ state was populated through non-radiative relaxation from the ^3^F_2,3_ states and later on depopulated directly to the ^3^H_6_ ground state, giving rise the emissions at 800 nm as shown in Figure 3b. Then, it can be concluded that the weaker emission at 785 nm in Figure 3a belongs to the ^1^G_4_ → ^3^H_5_ transition.

**Figure 4.**
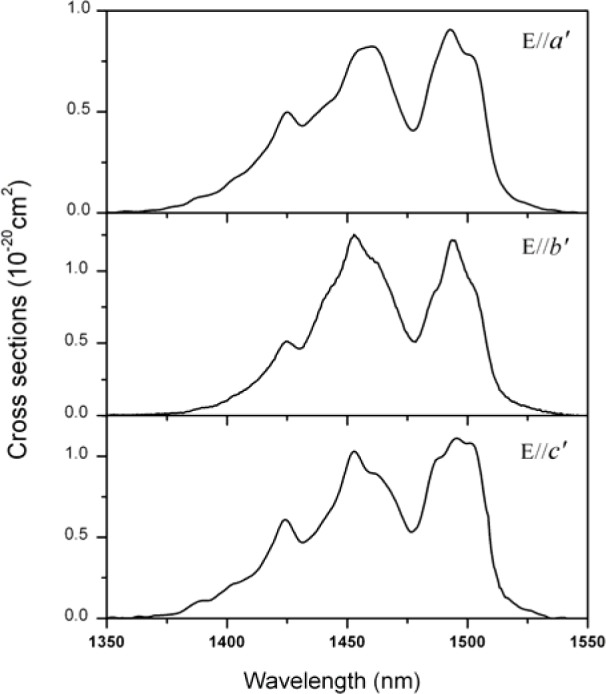
Polarized emission cross-sections of Tm^3+^:Li_3_Ba_2_Gd_3_(MoO_4_)_8_ crystal for the ^3^H_4_ → ^3^F_4_ transition around 1.5 μm.

The stimulated emission cross-sections of the ^3^H_4_ → ^3^F_4_ transition around 1.5 μm were calculated from the fluorescence spectra using the Füchtbauer-Ladenburg(F-L) formula [2]:

$$\sigma_{\text{em,q}}(\lambda )=\frac{\lambda^{5}{\text{A}}_{\text{q}}(\text{J}\to {\text{J}}^{\prime }){\text{I}}_{\text{q}}(\lambda )}{8\pi {\text{cn}}^{2}\int^{\text{​}}\lambda {\text{I}}_{\text{q}}(\lambda )\text{d}(\lambda )}$$ (2)

where c is the speed of light in the vacuum; I_q_(λ) is the relative fluorescence intensity at wavelength λ. Then, the emission cross-sections for three polarizations are shown in Figure 4. For E//*b*′, the peak emission cross-sections are 1.25 × 10^−20^ cm^2^ at 1452 nm. For E//*a*′ and E//*c′*, the peak emission cross-sections are located at 1495 nm and the values are 9.16 × 10^−21^ cm^2^ and 1.12 × 10^−20^ cm^2^, respectively. The ^3^H_4_ → ^3^F_4_ transition of the Tm^3+^ ions has provided a promising approach to achieve 1.5 μm lasers in a more efficient four-level laser operation scheme than the Er^3+^ ions. However, their practical applications at this wavelength are inevitably restricted by a detrimental bottlenecking effect because the lifetime of the ^3^F_4_ state is generally much longer than that of the ^3^H_4_ state. As a result, to achieve laser operation via ^3^H_4_ → ^3^F_4_ transition, some codopants, such as Ho^3+^, Yb^3+^, and Tb^3+^ should be introduced as deactivator to depopulate the ^3^F_4_ state [20].

**Figure 5.**
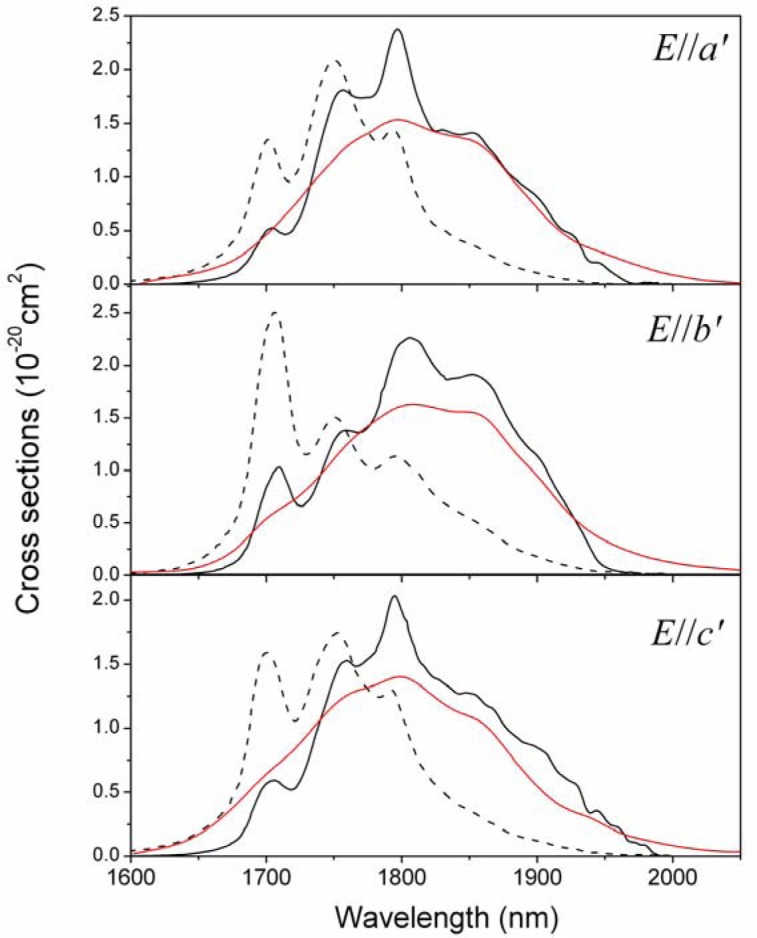
Polarized absorption (dot line), emission (solid line) cross-sections and emission spectra (red line) of Tm^3+^:Li_3_Ba_2_Gd_3_(MoO_4_)_8_ crystal for the^3^F_4_ → ^3^H_6_ transition.

Due to the restriction of the test conditions, only the uncalibrated emission spectra of the ^3^F_4_ → ^3^H_6_ transition, around 2 μm, were obtained, as shown in Figure 5. Thus, the emission cross-sections of this transition were calculated from the absorption spectra according to the reciprocity method [21]:

$$\sigma_{\text{em}}(\lambda )=\sigma_{\text{abs}}(\lambda )\frac{{\text{Z}}_{1}}{{\text{Z}}_{\text{u}}}\text{exp}[({\text{E}}_{\text{zl}}-\text{hc}/\lambda )/\text{kT}]$$ (3)

where k is the Boltzmann’s constant; Z_l_ and Z_u_ are the partition functions of lower and upper states, respectively; E_zl_ is the zero-line energy defined as the energy separation between the lowest Stark levels of the upper and lower multiplets. However, the precise energy level diagram of Tm^3+^ ions in Li_3_Ba_2_Gd_3_(MoO_4_)_8_ crystal is not available now. Therefore the values of Z_l_/Z_u_ and E_zl_ were roughly taken as Z_l_/Z_u_ = 1.42 and E_zl_ = 5600 cm^−1^ (0.694 eV), respectively, in agreement with those of the isostructural Tm^3+^:Li_3_Ba_2_Lu_3_(MoO_4_)_8_ crystal [16]. Then, the emission cross-sections are obtained and listed in Figure 5 combining with the corresponding absorption cross-sections. For all polarizations, the peak emission cross-sections are located at 1796 nm and the values are 2.38, 2.27, and 2.05 × 10^−20^ cm^2^ for E//*a*′, E//*b*′, and E//*c*′, respectively, which are larger than those of other disordered molybdate crystals (see Table 2). Furthermore, the FWHMs of the emission cross-sections are 133, 154 and 136 nm for E//*a*′, E//*b*′, and E//*c*′, respectively, which are similar to those of other disordered molybdate crystals in Table 2.

**Figure 6.**
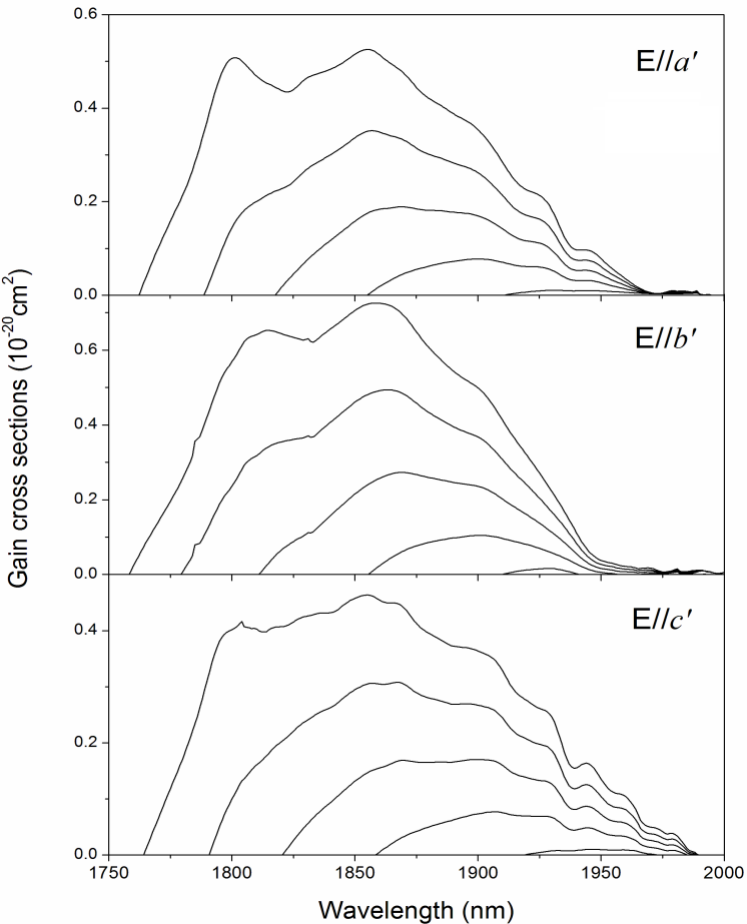
Gain cross-sections of Tm^3+^:Li_3_Ba_2_Gd_3_(MoO_4_)_8_ crystal for different values of inversion ratio β (β *=* 0.1, 0.2, 0.3, 0.4, 0.5).

From the absorption and emission cross-sections calculated by the reciprocity method, the gain cross-section, σ_g_, can be calculated according to the following equation:

$$\sigma_{\text{g}}=\beta \sigma_{\text{em}}-(1-\beta )\sigma_{\text{abs}}$$ (4)

where β is the ratio of the number of the excited Tm^3+^ ions to the total number of Tm^3+^ ions; σ_em_ and σ_abs_ are the emission and absorption cross-sections, respectively. The calculated gain cross-sections for several values of β are shown in Figure 6. According to Figure 6, a minimum inversion ratio of 0.1 is needed to achieve laser operations. For an inversion ratio of β *=* 0.3, a tunable range wider than 180 nm is possible for all polarizations.

**Figure 7.**
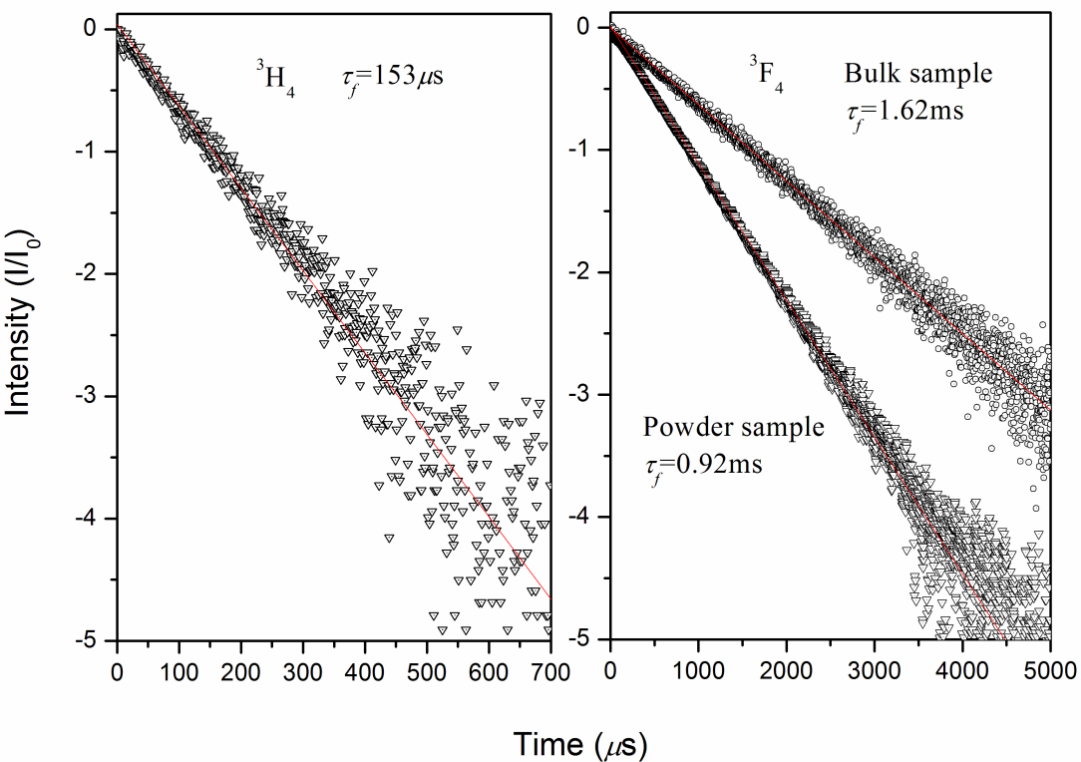
Fluorescence decay curves of ^3^H_4_ and ^3^F_4_ state.

The fluorescence decay curve of the ^3^H_4_ and ^3^F_4_ state was recorded by monitoring the emissions of the ^3^H_4_ → ^3^F_4_ and ^3^F_4_ → ^3^H_6_ transitions at 1500 and 1900 nm under excitation at 800 and 1730 nm, respectively. The decay curve of the ^3^H_4_ state exhibits a single exponential behavior as shown in Figure 7. The lifetime is obtained to be 153 μs by linear fitting, which is close to the radiative one. The high quantum efficiency η (τ_f_/τ_r_) of the ^3^H_4_ state means the cross-relaxation is inefficient in the crystal due to the low Tm^3+^ concentration. The lifetime of ^3^F_4_ state for the bulk crystal is measured to be 1.62 ms and much larger than the calculated radiative lifetime. Such a large discrepancy is mainly caused by the re-absorption effect, which has been widely observed in Tm^3+^-doped crystals [8,16]. To obtain the intrinsic lifetime of the ^3^F_4_ state, the powder method was adopted in this work [22]. A piece of bulk crystal was grounded into fine particles and diluted to a lower concentration of 0.25 at% (1.25 × 10^−19^ cm^2^) with the powder of pure Li_3_Ba_2_Gd_3_(MoO_4_)_8_ crystal. Then, the powder was immersed into ethylene glycol (EG), which was used as refractive index matching fluid to reduce the internal reflection within the particles. The fluorescence lifetime of the powder sample was measured to be 0.92 ms, in reasonable agreement with the radiative one calculated by the J-O theory.

## 4. Conclusions

A Tm^3+^:Li_3_Ba_2_Gd_3_(MoO_4_)_8_ crystal has been successfully grown by the top seeded solution growth (TSSG) method, and the detailed spectral properties of the crystal were characterized and investigated on the basis of the J-O theory. The main spectral parameters of the crystal are listed in Table 2 and compared with other Tm^3+^-doped crystals. It can be found that Tm^3+^:Li_3_Ba_2_Gd_3_(MoO_4_)_8_ crystal possesses similar spectral characters to other disordered molybdate crystals, *i.e.*, all of them exhibited broad optical bands, large absorption and emission cross-sections, as well as long lifetimes of ^3^F_4_ state. In summary, the main spectroscopic parameters of the Tm^3+^:Li_3_Ba_2_Gd_3_(MoO_4_)_8_ crystal are comparable to those of the Tm^3+^:Li_3_Ba_2_Lu_3_(MoO_4_)_8_ crystal. As a consequence, in view of the excellent laser performance of the Tm^3+^:Li_3_Ba_2_Lu_3_(MoO_4_)_8_ crystal [16], we may expect the Tm^3+^:Li_3_Ba_2_Gd_3_(MoO_4_)_8_ crystal to be a potential solid state laser material at ~2.0 μm.

## Figures and Tables

**Table 1.** The spontaneous emission probabilities, fluorescence branching ratios and radiative lifetimes of Tm^3+^:Li_3_Ba_2_Gd_3_(MoO_4_)_8_ crystal.

Transitions	λ_em_ (nm)	E//*a′*	E//*b′*	E//*c′*	β	τ_r_ (μs)

		A_ed_ + A_md_ (s^−1^)	A_ed_ + A_md_ (s^−1^)	A_ed_ + A_md_ (s^−1^)		
^1^G_4_ → ^3^H_6_	477	5035.7	5474.1	4258.7	0.56	113
^3^F_4_	649	345.8 + 13.8	580.8 + 13.9	388.7 + 12.8	0.05	
^3^H_5_	780	2031.8 + 207.2	2334.7 + 208.7	1936.4 + 192.4	0.26	
^3^H_4_	1170	993.1 + 47.3	925.3 + 47.7	839.2 + 44.03	0.11	
^3^F_3_	1485	93.2 + 3.8	173.7 + 3.9	113.2 + 3.6	0.02	
^3^F_2_	1635	27.9	59.8	33.4	0.004	

^3^H_4_ → ^3^H_6_	785	5544.6	5597.5	4792.5	0.90	170
^3^F_4_	1430	469.8 + 26.8	497.8 + 27.1	407.2 + 25	0.08	
^3^H_5_	2165	47.9 + 14	131.5 + 14.2	62.8 + 13	0.02	

^3^H_5_ → ^3^H_6_	1225	676.0 + 117	969.5 + 118	671.6 + 108.9	0.99	1110
^3^F_4_	3825	12.8 + 0.16	15.2 + 0.16	10.9 + 0.14	0.01	

^3^F_4_ → ^3^H_6_	1800	970.0	1040.0	791	1.00	1070

**Table 2.** The main spectral parameters of Tm^3+^:Li_3_Ba_2_Gd_3_(MoO_4_)_8_ and other Tm^3+^-doped crystals.

Properties	Li_3_Ba_2_Gd_3_(MoO_4_)_8_	LiGd(MoO_4_)_2_	LiLa(MoO_4_)_2_	BaGd_2_(MoO_4_)_4_	Li_3_Ba_2_Lu_3_(MoO_4_)_8_
λ_abs_ (nm)	796	795 (σ)	795	798	797 (*a*′)
	–	796 (π)	–	–	782 (*b*′)
	–	–	–	–	804 (*c*′)

σ_a_ (10^−20^ cm^2^)	4.08 (*a*′)	4.33 (σ)	4.04 (σ)	2.5 (X)	3.8 (*a*′)
	2.59 (*b*′)	1.59 (π)	1.53 (π)	3.5 (Y)	2.7 (*b*′)
	3.55 (*c*′)	–	–	2.1 (Z)	3.1 (*c*′)

FWHM (nm)	9 (*a*′)	8 (σ)	8 (σ)	7 (X)	8 (*a*′)
	11 (*b*′)	37 (π)	36 (π)	8 (Y)	12 (*b*′)
	8 (*c*′)	–	–	17 (Z)	8 (*c*′)

λ_e_ (nm)	–	1786 (σ)	1787 (σ)	1800 (X)	1800 (*a*′)
	1796	1838 (π)	1837 (π)	1805 (Y)	1812 (*b*′)
	–	–	–	1819 (Z)	1805 (*c*′)

σ_em_ (10^−20^ cm^2^)	2.38 (*a′*)	2.44 (σ)	1.48 (σ)	1.3 (X)	2.65 (*a*′)
	2.27 (*b′*)	2.07 (π)	1.61 (π)	1.8 (Y)	2.30 (*b*′)
	2.05 (*c′*)	–	–	1.3 (Z)	2.25 (*c*′)

FWHM (nm)	133 (*a*′)	175 (σ)	143 (σ)	110 (X)	90 (*a*′)
	154 (*b*′)	160 (π)	164 (π)	84 (Y)	168 (*b*′)
	136 (*c*′)	–	–	200 (Z)	137 (*c*′)

τ_f_ of ^3^F_4_ (ms)	0.92	0.93	1.29	–	0.97

References	This work	[8]	[6]	[7]	[16]
